# The envelope protein of Zika virus interacts with apolipoprotein E early in the infectious cycle and this interaction is conserved on the secreted viral particles

**DOI:** 10.1186/s12985-022-01860-9

**Published:** 2022-07-28

**Authors:** Yannick Tréguier, Jade Cochard, Julien Burlaud-Gaillard, Roxane Lemoine, Philippe Chouteau, Philippe Roingeard, Jean-Christophe Meunier, Marianne Maquart

**Affiliations:** 1 INSERM U1259 MAVIVH, Université de Tours et CHU de Tours, Tours, France; 2 Plateforme IBiSA des Microscopies, Université de Tours et CHU de Tours, Tours, France; 3 Plateforme B Cell Ressources, EA4245 T2I, Université de Tours, Tours, France

**Keywords:** Zika virus, Apolipoprotein E, Arbovirus, Virus/cell interaction, *Flaviviridae*, Electron microscopy

## Abstract

**Background:**

Zika virus (ZIKV), a member of the *Flaviviridae* family, has caused massive outbreaks of infection in tropical areas over the last decade and has now begun spreading to temperate countries. Little is currently known about the specific host factors involved in the intracellular life cycle of ZIKV. *Flaviviridae* viruses interact closely with host-cell lipid metabolism and associated secretory pathways. Another *Flaviviridae*, hepatitis C virus, is highly dependent on apolipoprotein E (ApoE) for the completion of its infectious cycle. We therefore investigated whether ZIKV also interacted with this protein.

**Methods:**

ZIKV infections were performed on both liver and microglia derived cell lines in order to proceed to colocalization analysis and immunoprecipitation assays of ApoE and Zika envelope glycoprotein (Zika E). Transmission electron microscopy combined to immunogold labeling was also performed on the infected cells and related supernatant to study the association of ApoE and Zika E protein in the virus-induced membrane rearrangements and secreted particles, respectively. Finally, the potential of neutralization of anti-ApoE antibodies on ZIKV particles was studied.

**Result:**

We demonstrated an interaction between ApoE and the Zika E protein. This specific interaction was observed in virus-induced host-cell membrane rearrangements, but also on newly formed intracellular particles. The partial neutralizing effect of anti-ApoE antibody and the immunogold labeling of the two proteins on secreted virions indicates that this interaction is conserved during ZIKV intracellular trafficking and release.

**Conclusions:**

These data suggest that another member of the *Flaviviridae* also interacts with ApoE, indicating that this could be a common mechanism for the viruses from this family.

## Introduction

Zika virus (ZIKV) is an arthropod-borne virus (arbovirus) from the *Flaviviridae* family first identified in Uganda in 1947 and brought to public attention by major outbreaks in the South Pacific area in 2007, 2013 and 2014 [1–4]. The most recent massive outbreaks in 2015–2016, with millions of cases and extending to Brazil, South and Central American countries, and the West Indies, led the WHO to declare ZIKV as a public health emergency [5, 6]. The transmission of the virus by mosquitoes of the genus *Aedes* occurs in most tropical regions worldwide, and the local transmission of ZIKV infection has also been reported in European countries and the USA, highlighting the increasing risk of future outbreaks of ZIKV infection in non-tropical areas [7–11].

ZIKV infection induces a broad range of symptoms ranging from arthralgia, rash, moderate fever, and conjunctivitis to neurological disorders, such as Guillain-Barré syndrome, myelitis, and encephalitis. It also causes microcephaly in the newborns of women infected during pregnancy [12]. There is currently no specific antiviral treatment for ZIKV infection, for which only symptomatic treatments are used. Furthermore, despite global efforts to develop a vaccine, none of those generated to date has proved effective enough for commercialization [13]. This situation highlights the need to increase our knowledge of the mechanisms underlying the pathophysiology of ZIKV infection and the basic ZIKV life cycle.

Several types of cells are involved in the ZIKV infection process. Indeed, the inoculum, which is delivered by mosquito bites, first infects skin cells, such as keratinocytes and fibroblasts, eventually reaching the skin-resident dendritic cells, which mediate its entry into the central nervous system (CNS) via the lymph nodes [14]. The infection of macrophages and CD14+ CD16+ monocytes in the lymph nodes, thus, leads to viremia and the spread of ZIKV to other organs, such as the placenta, testes, brain and liver [15–20].

Like other members of the *Flaviviridae* family, ZIKV induces host-cell membrane rearrangements and its intracellular life cycle is closely associated with lipid synthesis and the associated secretory pathways [21–24]. The translation of ZIKV RNA leads to the production of a polyprotein containing non-structural and structural ZIKV proteins that induces endoplasmic reticulum (ER) membrane rearrangements to generated so-called “convoluted membranes” (CMs) [25]. The infectious cycle of ZIKV has not been described in full but is likely to resemble that of other members of the *Flaviviridae* family that have been studied more extensively. Individual viral replication sites cluster together in the ER membranes to form vesicle packets (VPs) under the specific action of the NS4A protein [26]. Viral morphogenesis then occurs in the ER membrane domains juxtaposed against but separate from replication sites, in which ZIKV envelope glycoprotein (Zika E protein) from the ER lumen interacts with cellular chaperone proteins, such as calnexin, with the capsid proteins (C) located on the cytoplasmic side of the ER membrane [26–30]. The newly formed ZIKV particles presumably accumulate in an ER-Golgi intermediate compartment, in which Zika E protein undergoes its final glycosylation and proteolytic maturation. The virions in large membranous compartments may then be secreted via the direct fusion of these compartments with the plasma membrane or as individual virions after repackaging into individual small membranous vesicles [22, 31, 32]. The steps in the intracellular cycle have been described in part, but the host cellular factors involved in ZIKV replication, morphogenesis, and secretion process remain to be identified.

Hepatitis C virus (HCV), another member of the *Flaviviridae* family, is highly dependent on apolipoprotein E (ApoE) for the completion of its life cycle. Indeed, ApoE is crucial for the morphogenesis and secretion steps of the HCV life cycle. It interacts with both the structural envelope glycoproteins, E1 and E2, in the ER lumen, but also with non-structural protein 5A (NS5A) on diacylglycerol O-acyltransferase-1 (DGAT-1)-generated lipid droplets [33]. ApoE has also been identified as a component of the released HCV particles and has been shown to be engaged in immune system escape and entry into host cells [34–38]. This protein is naturally involved in many intra- and extracellular pathways, including the regulation of lipid metabolism and transport between the liver and peripheral cells, lipoprotein morphogenesis, lipid content homeostasis in plasma and tissues, the cellular global stress response, immune responses, and the formation and composition of several extracellular vesicles, such as exosomes [39–44]. Interestingly, ApoE is synthesized in many different organs and cell types, including the liver, brain, adrenal glands, testes, kidneys and macrophages, and this protein is also found as a freely circulating form in the bloodstream [43, 45, 46].

ZIKV has recently been shown to alter host-cell lipid composition significantly [24]. Thus, given the tropism of ZIKV to several cell types expressing ApoE, and the key role of this protein in the HCV life cycle, we investigated the potential interaction between ZIKV and ApoE. Using immunoprecipitation assays, confocal and transmission electron microscopy (TEM), we were able to demonstrate a specific interaction of the Zika E protein with ApoE in virus-induced rearranged membranes and newly formed viral particles. This interaction was conserved in the secreted virions, as shown by immunogold labeling on purified virions observed by TEM, and their partial neutralization by anti-ApoE antibodies (Abs). Our findings, thus, demonstrate that a member of the *Flaviviridae* family other than HCV interacts with ApoE.

## Materials and methods

### Cell lines

Vero-E6 cells (ATCC CRL-1586) are an immortalized cell line from green monkey kidney commonly used to produce ZIKV inoculum. Huh7.5 cells are a subclone of Huh7 cells isolated from a hepatocellular carcinoma, and HMC3 (Human Microglia Clone 3; ATCC CRL-3304) cells are an immortalized cell line from human microglia. All three cell lines are easy to infect with ZIKV and support the complete cycle of the virus, from its entry into the cell to the release of new viral particles [47]. The cells were cultured in Dulbecco’s modified Eagle’s medium (DMEM) containing 4.5 g/L D-glucose, 4 mM L-glutamine (Gibco), and supplemented with 100 U/mL penicillin, 100 µg/mL streptomycin (Gibco) and 10% fetal calf serum (Thermo Fisher Scientific), under an atmosphere containing 5% CO_2_.

### Asian lineage ZIKV production and titration

Zika virus Asian lineage strain ZK Mar2016 was provided by the Centre National de Reference des Arbovirus, Marseille, France. This strain was propagated by two passages on Vero-E6 cells and viral stocks were titrated on Vero-E6 cells, with a limiting dilution technique and the calculation of TCID50. The final viral stock was titrated at 4.64 × 107 TCID50/mL. ZIKV was also propagated on Huh7.5 cells for neutralization assays and its titer was 6.81 × 104 TCID50/mL.

### Immunofluorescence labeling for colocalization analysis

Huh7.5 and HMC3 cells were infected at a multiplicity of infection (MOI) of 1, cultured on glass coverslips until 6, 12, 24- or 48 h post-infection, and fixed by incubation with 80% acetone in PBS for 20 min at -20 °C. The coverslips were incubated for 1 h at room temperature (RT) with the following primary antibodies: mouse anti-flavivirus envelope protein 4G2 Abs at a dilution of 1:100 (D1-4G2-4-15, Sigma-Aldrich), goat anti-ApoE Abs at a dilution of 1:500 (ab947, Merck-Millipore), and rabbit anti-calnexin Abs at a dilution of 1:500 (MA5-32332, Thermo Fisher Scientific) in 5% BSA in PBS. The coverslips were washed four times in PBS and incubated with the corresponding secondary Abs, at a dilution of 1:2000 in 5% BSA in PBS for 1 h at RT: donkey anti-mouse IgG, donkey anti-rabbit IgG and donkey anti-goat IgG conjugated with Alexa Fluor 594, 647 and 488, respectively (A21203, A31573, A11055, Thermo Fisher Scientific). The coverslips were washed four times in PBS and mounted in 50% Fluoromount-G-DAPI, 50% Fluoromount-G (Invitrogen).

Confocal microscopy analysis was performed with an SP8 confocal microscope (Leica). For each set of conditions, we analyzed 30 z-stacks with the Imaris (Oxford Instruments) software colocalization analysis plugin to quantify the colocalization of the proteins of interest. This plugin provides a Pearson coefficient by applying the same threshold to each set of stacks. The Pearson coefficient obtained is the “Pearson coefficient within the region of interest” (ROI). It reflects the strength of colocalization and lies between −1 (anticolocalization) and + 1 (exclusive colocalization). The mean Pearson coefficients obtained for the various sets of conditions were compared in ANOVA tests implemented in Prism8 (Graphpad) software.

### Co-immunoprecipitation assays

Both cell lines were infected at an MOI of 1. Two days post-infection the cells were lysed in 1% Triton X-100, 2 mM EDTA in PBS and total protein was quantified with the Pierce BCA Protein Assay Kit (23225, Thermo Fisher Scientific). For the immunoprecipitation assay, goat anti-ApoE Abs (ab947, Millipore) or goat anti-gp120 (HIV1) as irrelevant Abs (ab21179, Abcam) were fixed on rec-protein G-Sepharose 4B-conjugated microbeads (Invitrogen) at a dilution of 1:250 in PBS by incubation for two hours at RT with shaking. The microbeads were then blocked by incubation for 30 min with 5% BSA in PBS that had been passed through a filter with 0.2 μm pores and incubated overnight at 4 °C with 250 μg of cell lysate. The microbeads were washed three times in 0.2% Triton X-100 in PBS and resuspended in Laemmli buffer containing 1% β-mercaptoethanol. The co-immunoprecipitation product was analyzed by Western blotting.

The immunoprecipitation samples were heated at 96 °C for 6 min and subjected to electrophoresis in MOPS buffer at 50 mA on a 12% acrylamide iD PAGE Gel (ID-WCRUB1-005, ID-PA0121-010, respectively, Eurogentec) and the protein bands were then transferred to polyvinylidene difluoride membranes (GE10600023, Merck-Millipore) in TGS buffer containing 5% methanol at 100 V. The membranes were blocked by incubation for 30 min, at RT, in TBS buffer supplemented with 5% BSA, 0.2% Tween 20. Primary mouse anti-Zika E protein Abs (BF-1176-76, Biofront Technologies), goat anti-ApoE (ab947, Merck-Millipore) and rabbit anti-β-actin (ab8227, Abcam) antibodies were added, and the membranes were incubated overnight at 4 °C with shaking. The membranes were then washed three times with 0.5% Tween 20 in TBS for 10 min per wash and incubated with the corresponding secondary Abs conjugated with HRP: donkey anti-mouse, donkey anti-rabbit and donkey anti-goat (ab6820, ab6802 and ab6885, respectively, Abcam) for 1 h at RT with shaking. The membranes were then subjected to a further three washes, for 10 min each, with 0.5% Tween 20 in TBS. Chemiluminescence was measured with an ImageQuant LAS 500 chemiluminescence CCD camera (GE HealthCare) after incubation for five minutes at RT with Supersignal West Pico PLUS or Supersignal West Femto (Thermo Fisher Scientific).

### Ultrastructural observations of ZIKV-infected cells by transmission electron microscopy

For imaging of the cellular membrane rearrangements characteristic of ZIKV infection and intracellular Zika virions, Huh7.5 and HMC3 cells were infected at an MOI of 10 for 48 h and were then fixed by incubation in 4% paraformaldehyde (PFA), 1% glutaraldehyde in 0.1 M phosphate buffer, pH 7.2. They were then washed in PBS, post-fixed by incubation for 1 h with 1% osmium tetroxide (Electron Microscopy Sciences) and dehydrated in a graded series of ethanol solutions. Cell pellets were embedded in Epon resin (Sigma-Aldrich), which was allowed to polymerize for 48 h at 60 °C. Ultrathin (90 nm) sections were cut, stained with 5% uranyl acetate and 5% lead citrate, and deposited on electron microscopy (EM) grids coated with collodion membrane. The sections were examined under a Jeol 1400 Plus transmission electron microscope equipped with a OneView Gatan digital camera driven by Digital Micrograph software for image acquisition and analysis (Amatek).

### Immunogold labeling of cryosections according to the method of Tokuyasu for immunoelectron microscopy

Cells were infected at an MOI of 10, harvested 2 days post-infection, and fixed by incubation for 2 h with 4% PFA, 0.1% glutaraldehyde in phosphate buffer (pH 7.6). The cells were collected by centrifugation at 2000 × g for 10 min and washed twice for 5 min each with PBS before a final centrifugation at 2000 × g for 10 min. The supernatant was removed, and the cell pellets were embedded in gelatin 12% and infused with 2.3 M sucrose overnight at 4 °C. We cut 90 nm ultra-thin cryosections at − 110 °C on a LEICA UC7 cryo-ultramicrotome. Sections were retrieved in a 2% methylcellulose/2.3 M sucrose mixture (1:1) and collected onto formvar/carbon-coated nickel EM grids. The gelatin was removed by heating at 37 °C, and the sections were incubated on drops of mouse anti-Zika E protein 4G2 and rabbit anti-ApoE (ab52607, Abcam) Abs diluted at 1:50 in PBS. After six washes of 2 min each in PBS, the grids were incubated on drops of PBS containing secondary Abs at a dilution of 1:30. For experiments on Huh7.5 cells, donkey anti-mouse IgG Abs conjugated with 12 nm gold particles, and donkey anti-rabbit IgG Abs conjugated with 6 nm gold particles (ab105277, ab105294, Abcam, respectively) were used. We used donkey anti-mouse Abs conjugated with 6 nm gold particles and donkey anti-rabbit Abs conjugated with 10 nm gold particles for experiments on HMC3 cells (DAG-80608/1, DAR 6-80806/1, Aurion). Grids were finally washed with six drops of PBS (2 min each), post-fixed in 1% glutaraldehyde and rinsed with three drops of distilled water. Contrasting was performed by incubating EM grids on drops of 2% uranyl acetate, 2% methylcellulose (1:10 mixture). The sections were imaged with a transmission electron microscope (TEM) at 120 kV (Jeol 1400 Plus).

### Immunogold labeling of secreted viral particles

Two days post-infection at an MOI of 10, the supernatants of infected Huh7.5 and HMC3 cells were concentrated by centrifugation on a 10% sucrose cushion for 16 h at 197,000 × g. Virus pellets were resuspended by overnight incubation in PBS and samples were then incubated on formvar/carbon coated nickel EM grids for 5 min at RT and washed 3 times on PBS drops, for 2 min each. The EM grids were incubated for 1 h at RT on drops containing primary mouse anti-Zika E protein 4G2 and rabbit anti-ApoE (ab947, Merck-Millipore) Abs diluted 1:50 in PBS. The grids were washed five times on PBS drops, for 2 min each, and were then transferred to drops of PBS containing the secondary gold-conjugated donkey anti-mouse IgG and donkey anti-rabbit IgG (6, 10 or 12 nm, depending on the various experiments) Abs at a dilution of 1:30, for incubation for 1 h at RT. The grids were washed three times, for 2 min each, in PBS, and then three times in distilled water. They were then fixed by incubation with 4% PFA, 1% glutaraldehyde, 0.1 M phosphate buffer (pH 7.2) at RT for 20 min. Contrasting staining was performed with 2.5% uranyl acetate in distilled water and the grids were observed under a TEM Jeol 1400 Plus.

### ZIKV neutralization assays with anti-ApoE and anti-Zika E protein Abs

Huh7.5 cells were infected at an MOI of 1 for 1 h. The supernatants were harvested 2 days post-infection and mixed with sucrose in Hepes (final sucrose concentration: 0.5 M) for freezing at − 80 °C before titration. We incubated 18,000 previously produced ZIKV particles with goat anti-ApoE (ab947, Merck-Millipore) or rabbit anti-Zika E protein monoclonal Abs (Ab02635-23.0, Sigma-Aldrich) at concentrations of 1.5 μg/mL and 10 μg/mL for 30 min at RT with shaking. Anti-goat and anti-rabbit isotype controls (AB-108-C, AB-105-C, R&D Systems) were used at a concentration of 50 μg/mL. Huh7.5 cells were then infected with the virus and incubated with the Ab mixture for 1 h and the medium was replaced. The infected cells were harvested 48 h post-infection and fixed by incubation for 20 min in 4% PFA. Cells were permeabilized by incubation for 20 min at RT in 0.2% Triton in PBS. The Zika E protein was targeted by incubation for 1 h with the anti-flavivirus envelope protein 4G2 Ab at a dilution of 1:200 in 0.1% Triton, 1% BSA in PBS. The primary Ab was detected with goat anti-mouse Ab conjugated to Alexa Fluor 488 (a32766, Invitrogen). Events were acquired on a FACS Melody flow cytometer (BD Biosciences), and the percentage of cells displaying positive staining was analyzed with FlowJo software (BD Biosciences). The results obtained from this assay were analyzed in ANOVA tests implemented in Prism8 (Graphpad) software.

## Results

### ApoE and Zika E protein colocalization occurs early and increases with time after infection

**Fig. 1:**
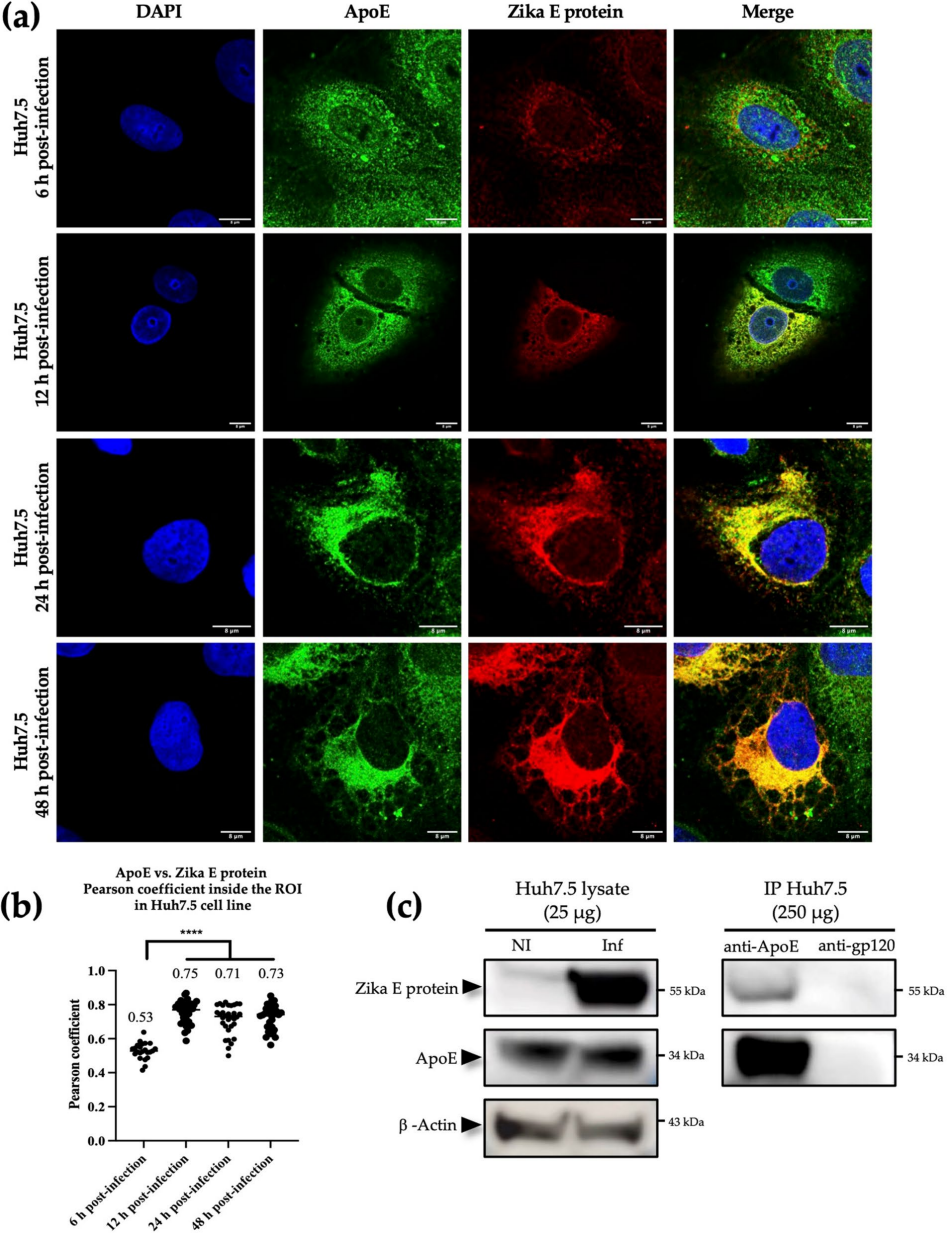
Kinetics of ApoE and Zika E protein colocalization and evidence of their interaction in infected Huh7.5 cells. **a** Kinetics of the colocalization of the ApoE and Zika E proteins, from 6 to 48 h post-infection at an MOI of 1. Scale bars = 8 µm. **b** Pearson coefficients for the colocalization of ApoE and Zika E protein within the ROI in Huh7.5 cells were performed on 30 z-stacks per condition. ****: *p* value < 0.0001 in ANOVA tests. **c** Co-immunoprecipitation of ApoE and Zika E protein. NI: naive cells; Inf: infected cells; IP: immunoprecipitation; anti-ApoE: goat anti-ApoE Abs; anti-gp120: anti-HIV-1 gp120 Abs used as an isotype control for the immunoprecipitation assay

Huh7.5 cells were infected at an MOI of 1 and the colocalization of the ApoE and Zika E proteins was studied by confocal microscopy 6-, 12-, 24- and 48-h post-infection. At 6 h post-infection, a faint but specific signal was detected for the Zika E protein, in the perinuclear area. The perinuclear and peripheral signal obtained for ApoE labeling was consistent with this protein being resident in the ER and trans-Golgi lumen, but also being a component of several cellular vesicles (Fig. 1A). At this time post-infection, both signals were present in the same area of the cell, but the ApoE signal appeared to be much stronger than the Zika E protein signal. The mean Pearson coefficient was comprised between 0.4 and 0.6 (0.53), indicating that the two proteins were only partially colocalized (Fig. 1B).

From 12 to 48 h post-infection, Zika E protein levels increased in infected cells, facilitating detection by fluorescence microscopy. The signal was located in the perinuclear area, extending to the periphery of the cells over time. At 24 h, an accumulation of Zika E protein was typically observed on one side of the infected cells, close to the nucleus, forming a pocket that was conserved 48 h post-infection (Fig. 1A). The ApoE signal in infected cells progressed from a diffuse signal at 6 h to a clustered signal at 48 h, and the signals of the two proteins were almost identical at 12-, 24- and 48-h post-infection (Fig. 1A). The signal remained diffuse in naïve cells (data not shown). The mean Pearson coefficients obtained for these conditions were 0.75, 0.71 and 0.73, respectively, all these values being significantly different from that of the Pearson coefficient obtained 6 h post-infection (*p value* < 0.0001). Thus, as soon as the Zika E protein is produced in a sufficient amount to be clearly detected by confocal microscopy it colocalizes with ApoE (Fig. 1B). The infected cells underwent massive morphological modifications during infection. Vacuole formation was observable by 6 h post-infection, and vacuoles increased in size between consecutive time points, affecting the subcellular distributions of the two proteins.

### Zika E protein interacts with ApoE in infected Huh7.5 cells

Huh7.5 cells were infected at an MOI of 1. Lysates were obtained from the infected cells and subjected to co-immunoprecipitation assays with anti-ApoE Abs at 48 h post-infection, with detection of the co-immunoprecipitated proteins by Western blotting. ApoE was detected as a 34 kDa band, of similar intensities in naïve and infected cells, whereas the Zika E protein was detectable only at a molecular weight of 55 kDa in infected cells (Fig. 1C). A strong signal at 34 kDa was observed when Abs targeting ApoE were used to immunoprecipitate ApoE, whereas irrelevant Abs (targeting the gp120 protein of HIV-1) gave no signal (Fig. 1C). Following ApoE immunoprecipitation, a band was detected at 55 kDa with anti-Zika E protein Abs, indicating that this protein was present in the co-immunoprecipitation product. The absence of a band at this molecular weight when anti-gp120 Abs were used confirmed the specificity of Zika E protein co-immunoprecipitation with ApoE (Fig. 1C). These data indicate that the ApoE and Zika E proteins interact in a specific manner in infected cells.

### ApoE-Zika E protein-complexes are located at ZIKV replication and morphogenesis sites in the ER of infected Huh7.5 cells

**Fig. 2:**
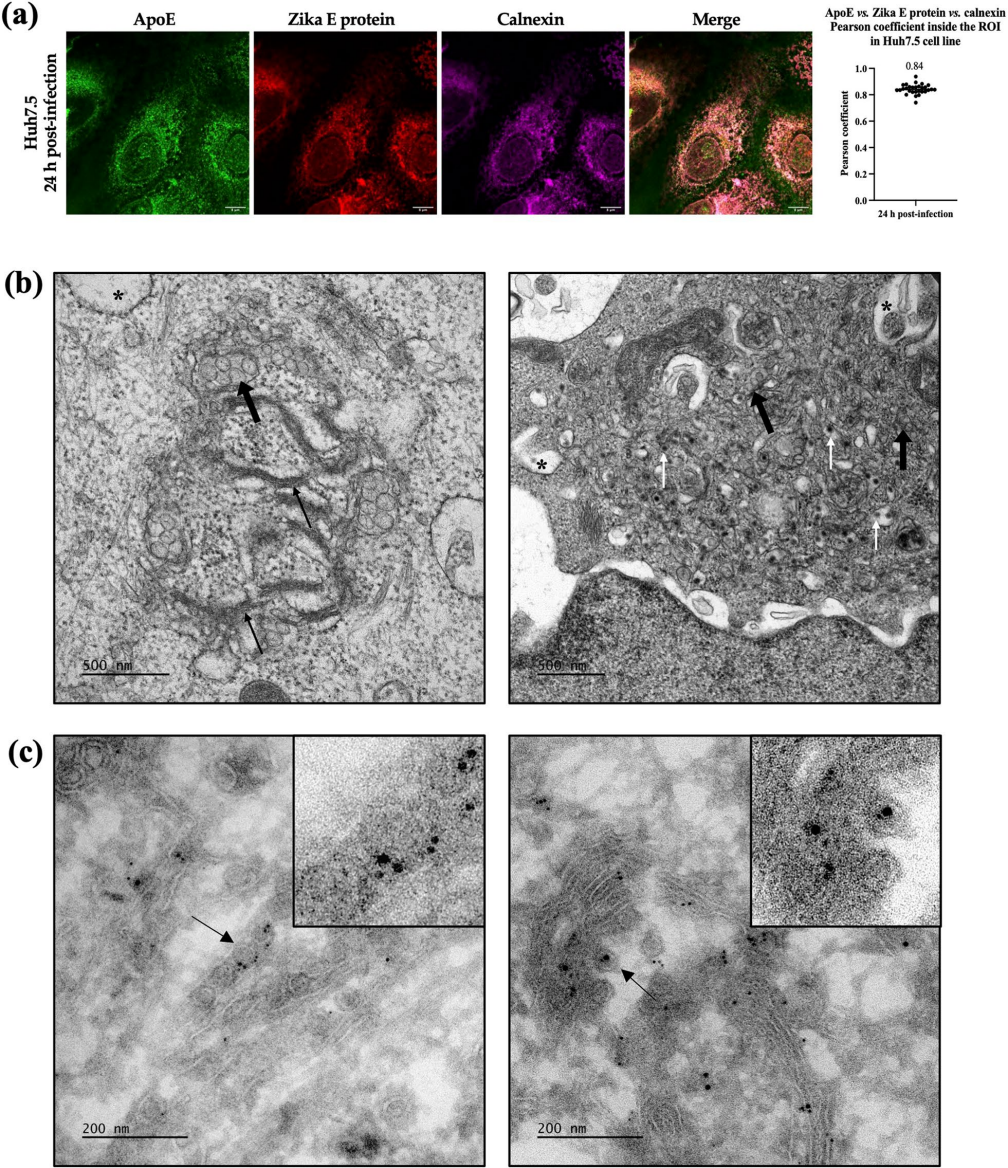
Detection of ApoE in ZIKV-induced ER membrane rearrangements in infected Huh7.5 cells. **a** Observation, by confocal microscopy, of the ApoE-Zika E protein channel with the ER marker calnexin in infected Huh7.5 cells and Pearson coefficient graph obtained from 30 z-stacks. Scale bars = 8 µm. **b** TEM observations of the characteristic membrane rearrangements induced by ZIKV infection in infected Huh7.5 cells. Thin black arrows: Zippered ER; thin white arrows: viral particles; bold black arrows: vesicle packets; asterisk: cytoplasmic vacuolization. Scale bars = 500 nm. **c** Immunogold labeling, on cryo-TEM sections, of ApoE and Zika E protein with 6 nm and 12 nm gold-conjugated Abs, respectively, in infected Huh7.5 cells. Insets show a high magnification of the area indicated by the thin arrow. Scale bars = 200 nm

The colocalization of ApoE-Zika E protein complexes with calnexin was analyzed in Huh7.5 cells 24 h post-infection at an MOI of 1. Calnexin is an ER-resident transmembrane protein and was, therefore, used as a marker of this compartment. The ApoE and Zika E protein colocalization signal was compared with the calnexin signal in infected cells. A strong immunofluorescence signal was obtained 24 h post-infection and calnexin labeling gave a clustered signal corresponding to the pockets shown in Fig. 1 (Fig. 2A). This signal overlapped strongly with the combined Zika E protein and ApoE signals, yielding a mean Pearson coefficient of 0.84, indicating a colocalization of these three proteins in ZIKV-infected Huh7.5 cells (Fig. 2A). The Zika E and ApoE proteins therefore appear to interact with each other in the ER. ZIKV replication and morphogenesis are known to occur in convolutions of the ER membranes [25]. We therefore tried to detect the two proteins of interest at such sites. Huh7.5 cells were infected at an MOI of 10 and examined by regular TEM at 48 h post-infection. Ultrastructural modifications characteristic of ZIKV infection [25, 47–49] were observed, with infected cells displaying massive cytoplasmic vacuolization (asterisk, Fig. 2B) and clusters of closely packed single-membrane vesicles known as VPs (bold black arrows, Fig. 2B), tightly associated with the ER. The ER of the infected cells was clearly modified into a specific form known as zippered ER (thin black arrows, Fig. 2B). Finally, newly synthesized ZIKV particles were visible in several infected cells (thin white arrows, Fig. 2B). Further cryo-TEM analysis was performed according to the Tokuyasu method with samples prepared in the same conditions of infection, for immunogold labeling of the Zika E and ApoE proteins. These two proteins were found to be both located on rearranged ER membranes and newly synthesized ZIKV particles (Fig. 2C).

### ApoE and Zika E proteins also interact in HMC3 cells and colocalize in virus-induced rearranged ER membranes in these cells

**Fig. 3:**
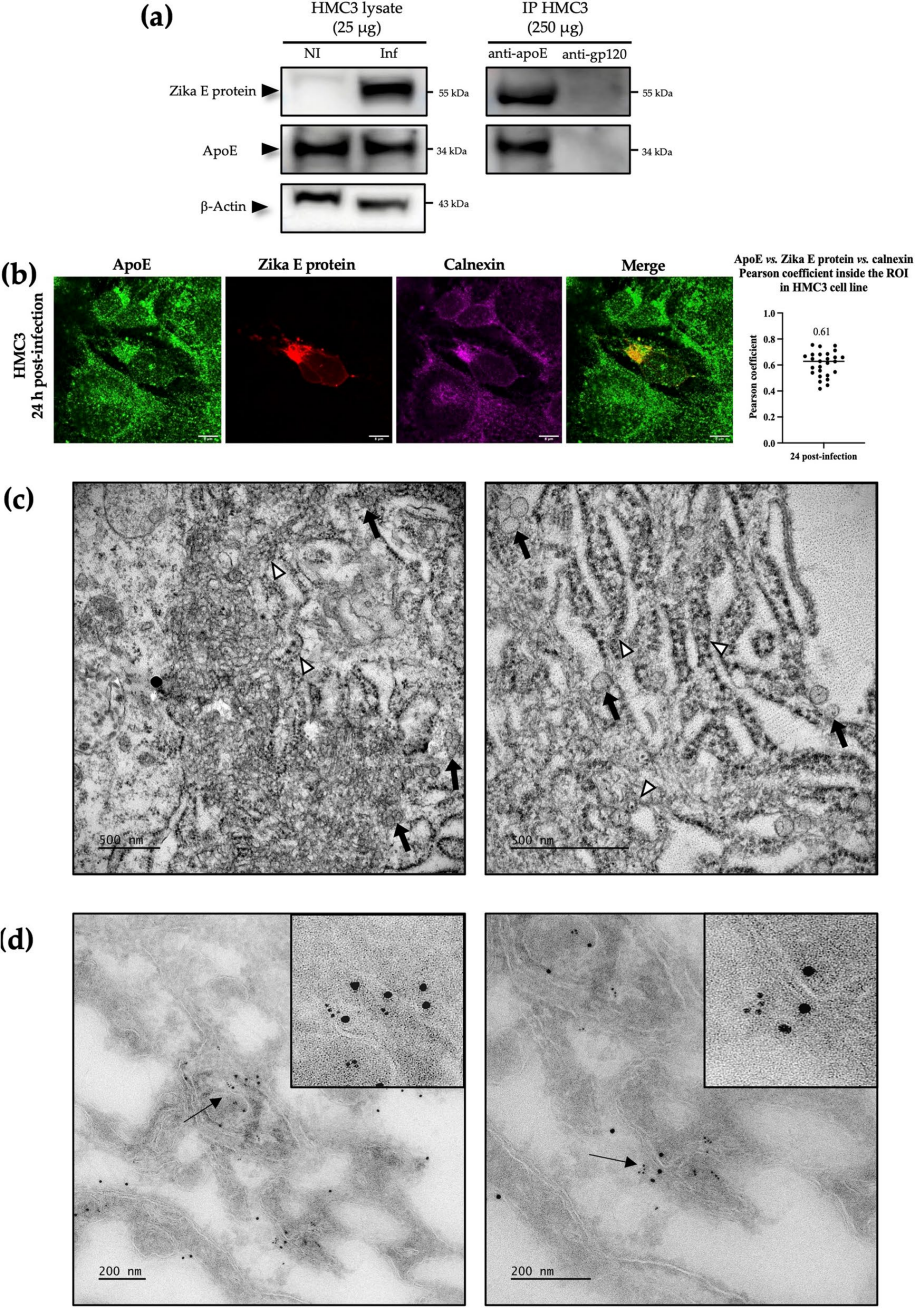
ApoE and Zika E protein interact in ZIKV-induced ER rearrangements in infected HMC3 cells. **a** Western-blot analysis of the co-immunoprecipitation of ApoE and Zika E protein after 48 h post-infection. NI: naive cells; Inf: infected cells; IP: immunoprecipitation; anti-apoE: goat anti-ApoE Abs; anti-gp120: anti-HIV-1 gp120 Abs used as an isotype control for immunoprecipitation assays. **b** Assay of the colocalization of the ApoE-Zika E proteins with calnexin after 24 h of infection; Pearson coefficient for colocalization within the ROI in HMC3 cells performed on 30 z-stacks. Scale bars = 8 µm. **c** Characteristic membrane rearrangements were observed in infected HMC3 cells by TEM. White triangles: convoluted membranes; bold black arrow: vesicle packets. Scale bars = 500 nm. **d** Immunogold labeling of ApoE and Zika E protein with 10 nm and 6 nm gold-conjugated Abs, respectively, in infected HMC3 cells analyzed on cryo-TEM sections. Scale bar = 200 nm

We checked that the results obtained in hepatocyte-derived Huh7.5 cells could be extended to a brain-derived cell type, by investigating the interactions between ApoE and Zika E proteins in HMC3 cells. This cell line is derived from human microglia and is, therefore, representative of the cells of one of the principal target organs of ZIKV. Anti-ApoE Abs specifically co-immunoprecipitated the Zika E protein, demonstrating an interaction between the ApoE and Zika E proteins in infected HMC3 cells 48 h post-infection (Fig. 3A). As in Huh7.5 cells, a weaker but positive colocalization of the two proteins was also observed with calnexin at 24 h post-infection. Indeed, the overlay of the ApoE-Zika E protein and calnexin channels gave a mean Pearson coefficient of 0.61 (Fig. 3B). Finally, TEM analysis was performed on HMC3 infected at an MOI of 10 at 48 h post-infection and revealed the existence of virus-induced membrane rearrangements in these cells (Fig. 3C). CMs (white triangles, Fig. 3C) and VPs (bold arrows, Fig. 3C) were observed, albeit in smaller numbers than in the infected Huh7.5 cell line. Nevertheless, immunogold labeling of the ApoE and Zika E proteins in cryo-TEM samples prepared according to the Tokuyasu method showed that these proteins were both located in these rearranged ER membranes as observed in ZIKV-infected Huh7.5 cells (Fig. 3D).

### ApoE protein is conserved on secreted ZIKV particles produced by both infected Huh7.5 and HMC3 cells

**Fig. 4:**
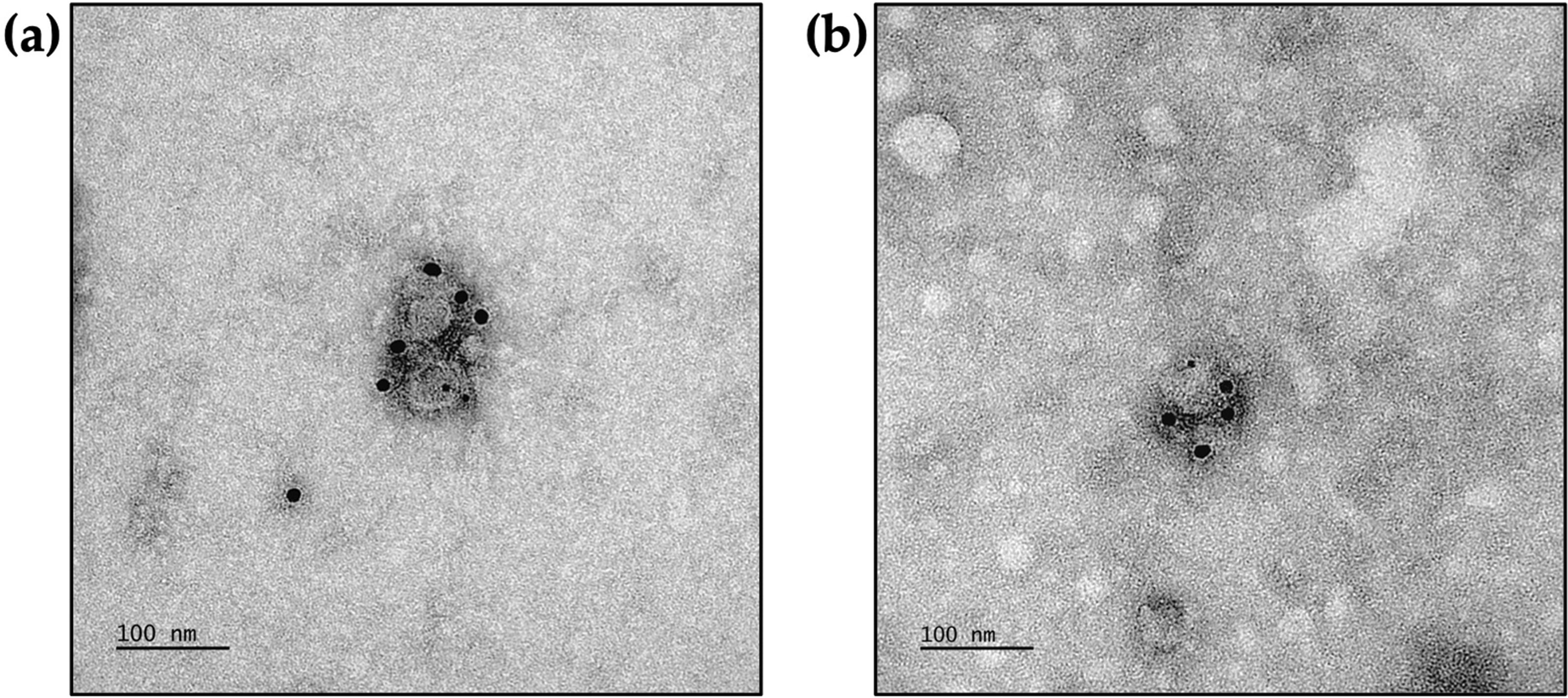
ApoE is conserved on ZIKV extracellular particles secreted from infected Huh7.5 and HMC3 cells. **a** Immunogold labeling of ApoE (6 nm) and Zika E protein (12 nm) on particles secreted by infected Huh7.5 cells and concentrated by centrifugation on a sucrose cushion. **b** The same procedure was applied to supernatant from infected HMC3 cells. ApoE and Zika E protein were labeled with 10 nm and 6 nm gold beads, respectively

We further investigated whether the intracellular interaction observed between ApoE and Zika E protein in Huh7.5 and HMC3 cells was conserved during secretory from the viral particles. We performed immunogold labeling for these two proteins on sucrose cushion-concentrated supernatants from the two cell lines 48 h post-infection at an MOI of 10. The viral particles released into the supernatant were labeled with gold beads binding to Zika E protein and ApoE attesting to the presence of both these proteins at the surface of the particles. These observations indicate that ApoE remains associated with the secreted particles (Fig. 4A and B).

**Fig. 5:**
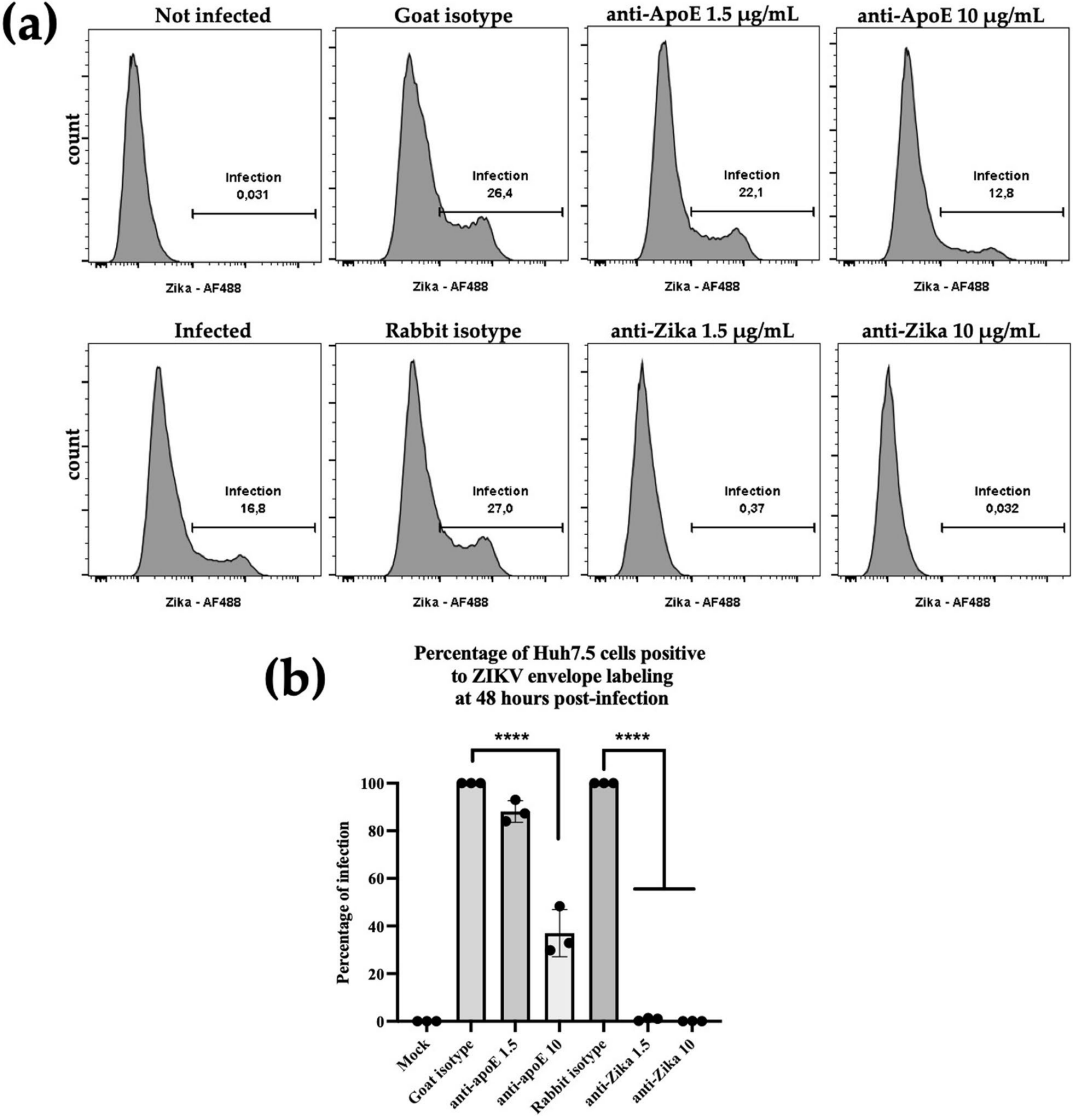
Neutralizing effect of anti-apoE Abs *vs* anti-Zika E protein Abs. **a** Example of FACS quantification of the number of cells positive for ZIKV envelope immunofluorescent labeling in the conditions tested. **b** Relative percentage of infected cells 48 h post-infection with particles previously incubated with various concentrations of Abs (indicated in μg/mL). The percentage of infection is relative to the corresponding isotype Abs. ****: *p* value < 0.0001 in ANOVA test

For confirmation of the presence of ApoE on secreted ZIKV particles by a different approach, we performed ZIKV infection neutralization assays on Huh7.5 cells, comparing anti-ApoE Abs with anti-Zika E protein Abs. Various concentrations of Abs were incubated with the viral particles prior the infection. Then, the infected cells were quantified by FACS after immunolabeling with anti-flavivirus envelope Abs 48 h post-infection. Representative results from a single experiment are shown in Fig. 5A. to illustrate the variability of the signal between conditions. On these graphs, the uninfected cells yield a single non-fluorescent peak, whereas the infected cells yield another, separate peak (Fig. 5A). The fluorescence intensity of this second peak was quantified to determine the number of ZIKV-infected cells in each set of conditions relative to naïve cells. A single non-fluorescent peak was obtained for cells not infected with ZIKV before the assay, validating our experiment protocol.

The neutralization abilities of both anti-ApoE and anti-Zika Abs were compared to their corresponding isotype, represented as the 100% of infection (Fig. 5B). Rabbit anti-Zika E protein Abs almost completely neutralized ZIKV particles infection when used at a concentration of 1.5 μg/mL, highlighted by a percentage of infection of ZIKV particles significantly decreased by 99% (*p* value < 0.0001). A similar result was obtained with rabbit anti-Zika E protein Abs at a concentration of 10 μg/mL (Fig. 5B). The anti-ApoE Abs provided a much lower level of neutralization at the same concentrations. Indeed, a mean of 88.1% of infection was observed when used at a 1.5 ug/mL concentration. However, at 10 μg/mL, the percentage of infection was significantly reduced by 60% indicating a neutralizing effect of the anti-ApoE Abs on the ZIKV infection process (*p* value < 0.0001). This dose-dependent inhibition indicates that anti-ApoE Abs partially inhibit ZIKV infection in Huh7.5 cells, confirming the presence of ApoE at the surface of ZIKV particles (Fig. 5B).

## Discussion

The landscape of host-cell factors involved in flavivirus life cycles can be deduced from the combination of specific and extrapolated observations for different members of the *Flaviviridae* family. ApoE has been shown to play a major role at various stages in the HCV infectious cycle [32–37], and intracellular lipid disorders have been reported during ZIKV infection [24]. In this study, we aimed to determine whether ApoE could also interact with ZIKV. We first demonstrated a partial colocalization of the ZIKV envelope protein and ApoE 6 h post-infection in Huh7.5 hepatic cells and then, a proper colocalization after 12 h, indicating the presence of the two proteins within the same cellular compartment as soon as Zika E protein was synthesized in sufficient amount. The E domain of the flavivirus polyprotein is known to be cleaved on the luminal side of the ER, resulting in an association of the E protein with the ER membrane [52]. This distribution was confirmed by the strong colocalization of ApoE, Zika E protein and calnexin, a marker of the ER compartment, 24 h post-infection. Beyond colocalization, a specific interaction between ApoE and Zika E protein was demonstrated by co-immunoprecipitation experiments on the two cell lines studied. These results were confirmed by immunogold labeling on cryo-TEM for both Huh7.5 and HMC3 cells. The ApoE and Zika E proteins were both located in the virus-induced membrane rearrangements described for flaviviruses and reported during ZIKV infection, in particular [25]. This feature was most apparent in Huh7.5 cells, which produced larger amounts of viral protein than HMC3 cells. In Huh7.5 cells, the ApoE and Zika E proteins were labeled in the CMs network and on newly formed viral particles. The association of ApoE with nascent viral particles suggests a preservation of the interaction between these two proteins during Zika E protein trafficking and maturation. This interaction may, therefore, occur early in Zika E protein synthesis in the ER lumen, as indicated by the partial colocalization observed 6 h post-infection, with ApoE translocated with Zika E protein to the ER cisternae in which viral morphogenesis occurs. As expected, given the lower levels of infection markers in HMC3 cells, the ultrastructural changes in this cell line were much more discreet than those in the Huh7.5 cell line. Nevertheless, we were able to identify several virus-induced structures, such CMs and VPs. Immunolabeling with gold beads of two different sizes demonstrated that the two proteins were located in these rearranged membranes. These results confirm that the observations reported above are not restricted to the Huh7.5 cell line and support the occurrence of ZIKV/ApoE interaction at the site of viral replication and morphogenesis. The differences observed in infectivity pattern between Huh7.5 and HMC3 cells may be due to different innate immune responses induced by ZIKV infection in these cells. Indeed, Huh7.5 cells have been described to be defective for several interferon-induced pathways allowing a better viral replication of viruses such as HCV as compared to regular Huh7 cells [53–55]. Also, the ZIKV infection pattern has been shown to be cell line dependent [56]. Overall, these data suggest that this interaction between the two proteins may persist in intracellular virus particles.

Conservation of the interaction of ZIKV with ApoE throughout the secretion pathway was demonstrated by the presence of ApoE on the surface of the secreted virions produced by the cells of both cell lines. Indeed, TEM analysis of purified virions with negative staining showed immunogold labeling of these virions with Abs against Zika E protein and ApoE. Furthermore, anti-ApoE Abs partly inhibited the entry of the virus into Huh7.5 cells. Anti-Zika E protein antibodies neutralized the infection much more efficiently, but the partial neutralization observed with anti-ApoE antibodies further confirms the presence of ApoE at the surface of the viral particles. This partial neutralization may result from steric hindrance due to the binding of the antibody to the surface of the particles.

Our findings, thus, suggest that the ZIKV envelope glycoprotein interacts with ApoE, as has already been shown for the HCV envelope proteins. However, the consequences of this interaction are unlikely to be the same for these two viruses from the *Flaviviridae* family. Indeed, for HCV, ApoE has been shown to be an essential host cell factor for the formation of infectious viral particles and for viral entry into cells, through its binding to coreceptors, such as scavenger receptor class B type I (SR-BI) and the low density lipoprotein receptor (LDL-R) [57]. However, the HCV infectious cycle is strictly limited to liver cells, whereas ZIKV has a much broader cell tropism. For example, ZIKV replicates efficiently in vitro in Vero cells, a monkey kidney cell line in which HCV cannot replicate. Interestingly, It has been shown that HCV can complete its infectious cycle in Vero cells provided that these cells are modified to express microRNA122 (mir-122), but also, and especially, ApoE and SR-BI [58]. This suggests that an association of *Flaviviridae* envelope proteins with ApoE may be a mechanism common to several members of this family, but particularly effectively exploited by HCV due to its strict liver-cell tropism. It is also possible that such an association is beneficial to the infectious cycles of other viruses, from other families, as the envelope of hepatitis B virus (HBV), another virus displaying strict liver tropism, was also recently shown to interact with ApoE [50, 51, 59]. As for HCV, this interaction appears to be necessary for both the formation of the HBV particle and its infectivity in liver cells. For ZIKV, there is currently no published evidence to suggest that this association is beneficial in ApoE-expressing cells. We cannot rule out the possibility that ApoE is involved in entry processes, given the partial neutralization observed with Abs targeting ApoE, but further investigations of this aspect are required. Moreover, during this study, we conducted ApoE-silencing assays in Huh7.5 cells, but this silencing had no effect on virus secretion (data not shown). Of note, a major difference occurs between HCV and other flaviviruses, as only HCV seems to be able to form a lipoviroparticle. However, in the case of HCV, the envelope proteins are not the only viral proteins to interact with ApoE since the non-structural protein NS5A interacts with apoE on the surface of lipid droplets, contributing to the morphogenesis of the HCV lipoviroparticle. Thus, the interaction between the viral envelope proteins and ApoE could be a common feature of different flaviruses, further complemented by the interaction of other proteins in the case of HCV for the formation of a lipoviroparticle. Nevertheless, our work suggests that the association between viral envelope proteins and ApoE may be a common mechanism exploited by some viruses, such as HCV and HBV, to optimize their infectious cycle.

## Data Availability

All data generated or analyzed during this study are included in this published article.

## Abbreviations

ZIKV: Zika virus
ApoE: Apolipoprotein E
Zika E: Zika envelope glycoprotein
CNS: Central nervous system
VPs: Vesicle packets
CMs: Convoluted membranes
ER: Endoplasmic reticulum
HCV: Hepatitis C virus
DGAT-1: Diacylglycerol O-acyltransferase-1
TEM: Transmission electron microscopy
DMEM: Dulbecco’s modified Eagle’s medium
MOI: Multiplicity of infection
ROI: Region of interest
RT: Room temperature
HBV: Hepatitis B virus
LDL-R: Low density lipoprotein receptor
SR-BI: Scavenger receptor class B type I
